# The RNA-binding protein Musashi2 governs osteoblast-adipocyte lineage commitment by suppressing PPARγ signaling

**DOI:** 10.1038/s41413-022-00202-3

**Published:** 2022-03-17

**Authors:** Jinlong Suo, Sihai Zou, Jinghui Wang, Yujiao Han, Lingli Zhang, Chenchen Lv, Bo Jiang, Qian Ren, Long Chen, Lele Yang, Ping Ji, Xianyou Zheng, Ping Hu, Weiguo Zou

**Affiliations:** 1 Department of Orthopedic Surgery and Institute of Microsurgery on Extremities, Shanghai Jiaotong University Affiliated Sixth People’s Hospital, 200233 Shanghai, China; 2 Chongqing Key Laboratory of Oral Diseases and Biomedical Sciences, Chongqing Municipal Key Laboratory of Oral Biomedical Engineering of Higher Education, Stomatological Hospital of Chongqing Medical University, 401147 Chongqing, China; 3 State Key Laboratory of Cell Biology, Shanghai Institute of Biochemistry and Cell Biology, CAS Center for Excellence in Molecular Cell Sciences, Chinese Academy of Sciences, University of Chinese Academy of Sciences, 200031 Shanghai, China; 4 Guangzhou Laboratory, No. 9 XingDaoHuan Road, Guanghzou International Bio lsland, 510005 Guangzhou, China; 5 Colorectal Cancer Center/Department of Gastrointestinal Surgery, Shanghai Tenth People’s Hospital Affiliated to Tongji University, Shanghai, China; 6 Institute for Stem Cell and Regeneration, Chinese Academy of Sciences, 100101 Beijing, China; 7 Bio-Research Innovation Center, Shanghai Institute of Biochemistry and Cell Biology, Suzhou, China

**Keywords:** Osteoporosis, Bone

## Abstract

Osteoporosis caused by aging is characterized by reduced bone mass and accumulated adipocytes in the bone marrow cavity. How the balance between osteoblastogenesis and adipogenesis from bone marrow mesenchymal stem cells (BMSCs) is lost upon aging is still unclear. Here, we found that the RNA-binding protein Musashi2 (*Msi2*) regulates BMSC lineage commitment. *Msi2* is commonly enriched in stem cells and tumor cells. We found that its expression was downregulated during adipogenic differentiation and upregulated during osteogenic differentiation of BMSCs. *Msi2* knockout mice exhibited decreased bone mass with substantial accumulation of marrow adipocytes, similar to aging-induced osteoporosis. Depletion of *Msi2* in BMSCs led to increased adipocyte commitment. Transcriptional profiling analysis revealed that *Msi2* deficiency led to increased PPARγ signaling. RNA-interacting protein immunoprecipitation assays demonstrated that *Msi2* could inhibit the translation of the key adipogenic factor *Cebpα*, thereby inhibiting PPAR signaling. Furthermore, the expression of *Msi2* decreased significantly during the aging process of mice, indicating that decreased *Msi2* function during aging contributes to abnormal accumulation of adipocytes in bone marrow and osteoporosis. Thus, our results provide a putative biochemical mechanism for aging-related osteoporosis, suggesting that modulating *Msi2* function may benefit the treatment of bone aging.

## Introduction

Aging-induced osteoporosis is characterized by reduced bone formation and the accumulation of adipocytes in the bone marrow chamber.^1,2^ Both osteoblasts and adipocytes are differentiated from the same multipotent precursor bone marrow mesenchymal stem cells (BMSCs).^3–5^ Compared with young MSCs, MSCs in old organisms show enhanced senescence, have reduced self-renewal and mainly differentiate into adipocytes instead of osteoblasts.^6^ The dynamic balance of MSC differentiation between adipogenesis and osteoblastogenesis is controlled by the expression of key transcription factors, including PPARγ, C/EBPα, and RUNX2.^7,8^ The adipose tissue of mice lacking C/EBPα is underdeveloped, and endogenous PPARγ cannot be induced.^9^ The transcription factor PPARγ plays a crucial role in bone development by inducing adipogenesis and inhibiting osteoblastogenesis.^10,11^ In addition to transcriptional regulation, posttranscriptional mechanisms also play important roles in regulating cell fate determination.^12^

RNA-binding proteins have been shown to regulate multiple steps of post-translational regulatory processes, such as RNA stability, RNA polyadenylation and translation, and determine cell fate.^13^ Whether RNA-binding proteins can regulate the commitment of MSCs has not been fully explored. Furthermore, the relationship between RNA-binding proteins and master transcription factors has not been fully elucidated. Revealing the functions of more RNA-binding proteins will help us further understand the orchestrated regulation of cell fate determination.

The Musashi (*Msi*) family of RNA-binding proteins contains two members, namely, *Msi1* and *Msi2*, in mammals.^13^ *Msi1* and *Msi2* are evolutionarily conserved, containing two tandem RNA recognition motifs and a carboxyl terminal poly-A–binding protein association domain.^13–15^ *Msi* proteins bind to r(G/A)U_1–3_AGU sequences (MSI binding elements, MBEs) at the 3′ untranslated region (UTR) of the target mRNA to prevent poly-A binding protein from entering the extension initiation complex to repress translation.^14,16^ Several studies have reported that MSI proteins act as translation repressors.^17–19^ MSI proteins contribute to the control of symmetric and asymmetric stem cell division, regulate stem cell function, and play a role in cell fate determination.^15,20^ *Msi1* is mainly involved in regulating the self-renewal of neuronal stem cells, and *Msi2* is mainly expressed in hematopoietic stem cells and regulates hematopoietic function.^15,21^ *Msi2* plays an important role in hair regeneration, maintaining the resting state of hair follicle stem cells, translation of cancer stem cells, and self-renewal and differentiation of hematopoietic stem cells.^22–24^ *Msi2* plays a critical role in the differentiation of osteoclasts in vitro, which are derived from HSCs. Loss of *Msi2* inhibits Notch signaling during osteoclast differentiation and induces apoptosis in preosteoclasts.^25^ Studies have found that Hh signaling can negatively regulate osteogenic differentiation by inhibiting RNA binding to *Msi1.*^26^ The Msi family plays a role in a variety of stem cells. Whether *Msi2* can regulate mesenchymal stem cells and whether it can regulate the fate determination of MSCs have not yet been reported. Whether *Msi2* has a regulatory effect on bone homeostasis and bone aging in vivo and the downstream molecular mechanism of the regulation are still unclear. The link between the RNA-binding protein *Msi*2 and osteoporosis is worth exploring in depth.

Here, we revealed the functions of *Msi2* in balancing the osteoblast/adipocyte lineage commitment of BMSCs and aging-induced osteoporosis. *Msi2* knockout mice displayed accumulation of adipocytes in the bone marrow cavity and decreased bone mass, mimicking osteoporosis. *Msi2* promotes the differentiation of BMSCs into osteoblasts and inhibits the differentiation of BMSCs into adipocytes. *Msi2* specifically binds the 3′UTR of mRNA of the key adipogenesis-related transcription factor *Cebpα* to inhibit its translation, thereby inhibiting PPARγ signaling. Furthermore, we found that *Msi2* expression was decreased in aged BMSCs, indicating that the decreased *Msi2* expression during aging shifts the osteogenesis/adipogenesis balance toward adipogenesis and leads to osteoporosis. Overall, these results suggested that increasing *Msi2* function may benefit the treatment of aging-related bone loss.

## Results

### The *Msi*2 expression level decreases during adipogenesis and increases during osteogenesis of BMSCs

**Fig. 1:**
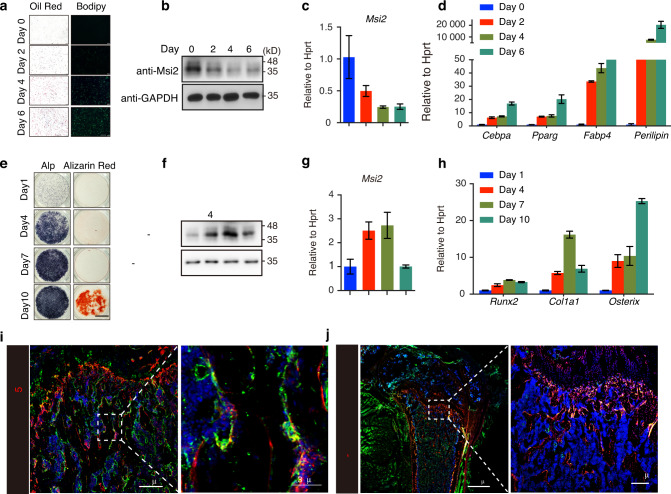
Changes in *Msi2* expression levels in the adipogenesis and osteogenesis of BMSCs. **a** BMSCs isolated from 4-week-old wild-type mice. Cultures were stained with Oil Red O and BODIPY as shown. Scale bar = 200 μm. **b** Western blot analysis of *Msi2* levels during adipogenesis for different durations. **c** qPCR analysis of *Msi2* expression in BMSCs during adipogenesis for the indicated durations. Data represent the mean ± SD, *n* = 4. **d** qPCR analysis of the expression of adipocyte markers, including *perilipin, Fabp4, Pparγ* and *Cebpα*, in BMSCs during adipogenesis for the indicated durations. Data represent the mean ± SD, *n* = 4. **e** BMSCs isolated from 6-week-old wild-type mice. Cultures were stained with ALP and Alizarin red S as shown. Scale bar = 3 mm. **f** Western blot analysis of *Msi2* levels during osteogenesis for different durations. **g** qPCR analysis of *Msi2* expression in BMSCs during osteogenesis for the indicated durations. Data represent the mean ± SD, *n* = 4. **h** qPCR analysis of the expression of osteoblast markers, including *Runx2*, *Col1a1* and *Osterix*, in BMSCs during osteogenesis for the indicated durations. Data represent the mean ± SD, *n* = 4. **i** Immunostaining of *Msi2* (green), CD105 (red) and DAPI (blue) in tibia from 6-week-old WT mice. Scale bar = 200 μm (left). Scale bar =30 μm (right). **j** Immunostaining of *Msi2* (green) and DAPI (blue) in femurs from 6-week-old Prx1-Cre Tdtomato mice. Scale bar = 1 000 μm (left). Scale bar = 200 μm (right)

BMSCs were able to differentiate into both osteoblasts and adipocytes. To explore the functions of *Msi*2 in BMSCs, we first surveyed the protein level of *Msi*2 during BMSC differentiation. When BMSCs were induced to differentiate into adipocytes that were stained with Oil Red O and BODIPY (Fig. 1a), both the mRNA and protein levels of *Msi*2 decreased, while the expression levels of adipocyte markers such as CCAAT/enhancer binding protein α (*Cebpα*), peroxisome proliferative activated receptor γ (*Pparγ*), fatty acid binding protein 4 (*Fabp4*) and *perilipin* increased, indicating efficient differentiation into adipocytes (Fig. 1b–d). In contrast, when BMSCs were induced to differentiate into osteoblasts that were stained with ALP and Alizarin red S (Fig. 1e), both the mRNA and protein levels of *Msi2* increased during the differentiation process. Consistently, the expression levels of osteoblast markers, including Runt-related transcription factor 2 (*Runx2*), Sp7 transcription factor (*Osterix*), and Collagen type 1 alpha 1 (*Col1α1*), increased, suggesting efficient differentiation into osteoblasts (Fig. 1f–h). The dynamic changes in *Msi2* expression levels during BMSC adipogenesis and osteogenesis indicate that *Msi2* may play distinct roles in adipogenesis and osteoblastogenesis from BMSCs.

We next determined the MSI2 expression level in long bone in vivo and found that MSI2 was highly expressed in the growth plate and trabecular bone (Fig. S1A). Interestingly, MSI2 was also expressed in the internal and external periostea but was barely expressed in cortical bone (Fig. S1B). CD105 is a marker of MSCs. Further research found that MSI2 and CD105 can be colocalized (Fig. 1i). Further research was performed to determine whether MSI2 is expressed in Prx1-positive cells, which are mainly MSCs. We found that MSI2 expression colocalized with Prx1-positive cells (Fig. 1j). These results further suggested that *Msi2* may have functions in MSC commitment and bone formation.

### *Msi2*-deficient mice display increased bone marrow adipocytes and decreased bone mass

**Fig. 2:**
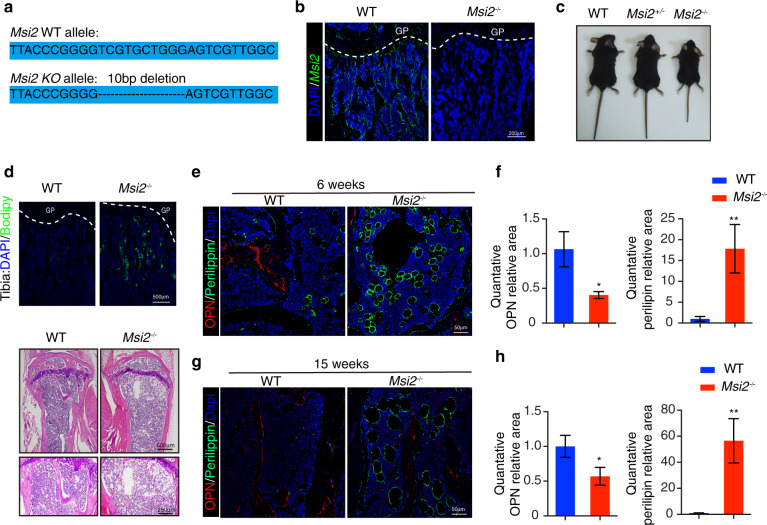
*Msi2*-deficient mice show increased bone marrow adipocytes. **a** Mouse construction strategy. **b** Immunostaining of MSI2 (green) and DAPI (blue) in tibiae from 6-week-old WT and *Msi2*^−*/*−^ mice. Scale bar = 200 μm. **c** Representative view of the wild-type, *Msi2*^*+/−*^, and *Msi2*^*−/*−^ 6-week-old mice. **d** BODIPY (green) staining of tibiae from the 6-week-old WT and *Msi2*^*−/−*^ mice. Scale bar = 500 μm (top). Hematoxylin-eosin staining of femurs from the 30-week-old WT and *Msi2*^*−/*−^ mice. Scale bar = 500 μm (middle). Scale bar = 500 μm (down). **e** Immunostaining of perilipin A/B (green) and OPN (red) of femurs from the 6-week-old WT and *Msi2*^−*/*−^ mice. Scale bar = 50 μm. **f** Quantification of the relative areas of OPN and perilipin in (**e**). **g** Immunostaining of perilipin A/B (green) and OPN (red) of femurs from the 15-week-old WT and *Msi2*^*−/−*^ mice. Scale bar = 50 μm. **h** Quantification of the relative areas of OPN and perilipin in (**g**)

To investigate the function of *Msi2* in BMSC differentiation, we generated *Msi2* knockout mice using CRISPR-Cas9 technology to introduce a frameshift in the first intron of *Msi2* (Fig. 2a). Immunofluorescence staining and western blotting confirmed the knockout of *Msi2* in bone and BMSCs (Fig. 2b and Fig. 6j). We tested the knockout efficiency of *Msi2* in the main organs of the knockout mice. The results showed that *Msi2* was almost completely eliminated in the *Msi2* knockout mice (Fig. S2A, B). In addition, we tested whether *Msi1*, a homolog of *Msi2*, has a compensatory effect in knockout mice, and the results showed that *Msi1* expression in the BMSCs of knockout mice was not significantly different from that in the control mice. (Fig. S2C). The *Msi2*^*−/−*^ mice survived normally after birth and had normal fertility. However, the *Msi2*^*−/−*^ mice exhibited short stature and skeletal dysplasia regardless of sex (Fig. 2c and Fig. S2D). Compared with the control mice, the Msi2 knockout mice had reduced body weight, body length, and femur length (Fig. S2E-G). BODIPY staining results of the *Msi2*^*−/−*^ mouse tibia revealed increased adipocyte accumulation in the tibia bone marrow of the *Msi2*^*−/−*^ mice, and older Msi2 knockout mice had more fat vacuoles in the bone marrow cavity (Fig. 2d). Immunofluorescence staining of *perilipin A*, a mature adipocyte marker, also confirmed adipocyte accumulation in the *Msi2*^−*/*−^ mice (Fig. 2e, f). Both the number and the size of adipocytes in the bone marrow cavity increased with age in the *Msi2*^*−/−*^ mice (Fig. 2g, h).

**Fig. 3:**
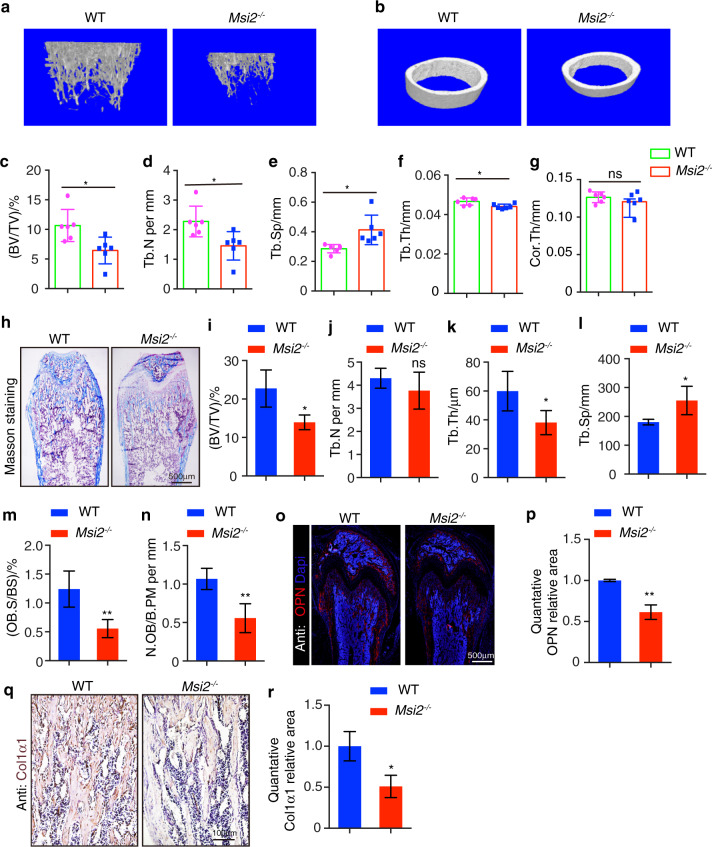
*Msi2*-deficient mice show decreased bone mass. **a** Three-dimensional μ-CT images of trabecular bone of distal femurs isolated from the 6-week-old female WT and *Msi2*^*−/−*^ mice (*n* = 6). **b** Three-dimensional μ-CT images of cortical bone of distal femurs isolated from the 6-week-old female WT and *Msi2*^−/−^mice (*n* = 6). **c–g** μ-CT analysis of distal femurs from the 6-week-old WT and *Msi2*^−*/*−^ mice for trabecular bone volume per tissue volume (BV/TV) (**c**), trabecular number (Tb.N) (**d**), trabecular separation (Tb.Sp) (**e**), trabecular thickness (Tb.Th) (**f**) and cortical bone thickness (Cor.Th) (**g**). **h** Masson trichrome staining of the 6-week-old WT and *Msi2*^−*/*−^ mice. Scale bar = 500 μm. **i–n** Histomorphometric analysis of distal femurs from the 5-week-old WT and *Msi2*^−*/−*^ mice to determine the trabecular bone volume per tissue volume (BV/TV) (**i**), trabecular number (Tb.N) (**j**), trabecular thickness (Tb.Th) (**k**) trabecular separation (Tb.Sp) (**l**) and number of osteoblasts per bone perimeter (N.Ob/B.Pm) (**m**) and osteoblast surface per bone surface (Ob.S/BS) (**n**). Data represent the mean ± SD, *n* = 4. *P < 0.05, **P < 0.01, ns indicates no significance, unpaired Student’s *t* test. **o** Immunostaining of OPN (red) and DAPI (blue) in femurs from the 6-week-old WT and *Msi2*^*−/*−^ mice. Scale bar = 500 μm. **p** Quantification of the relative area of OPN in (**o**). **q** Immunohistochemical staining of Col1α1 from the 6-week-old WT and *Msi2*^*−/−*^ mice. Scale bar = 100 μm. **r** Quantification of the relative area of Col1α1 in (**q**)

We further investigated whether bone formation was affected. Microquantitative computed tomography (μ-CT) analysis was performed to compare the changes in bone-related elements in the long bones of the *Msi2* knockout mice and the WT littermates. We found that the 6-week-old *Msi2*^−*/−*^ mice showed significantly decreased bone mass (Fig. 3a). Trabecular bone per tissue volume (BV/TV) in the *Msi2*^*−/−*^ mice was decreased compared to that in the age-matched WT littermates (Fig. 3c), accompanied by a reduction in trabecular number (Tb.N) (Fig. 3d), a reduction in trabecular bone thickness (Tb.Th) and an increase in trabecular bone spacing (Tb.Sp) (Fig. 3e, f). There was no significant difference in cortical bone thickness (Cor.Th) of the *Msi2*^*−/*−^ mice compared with that of the WT mice, which is consistent with the observation that *Msi2* is rarely expressed on cortical bone (Figs. 1j, 3b, g).

To further explore the function of *Msi2* in bone formation, we performed histomorphometric analysis to evaluate static and dynamic parameters of bone formation and resorption (Fig. 3h). Consistent with the μ-CT data, histomorphometric analysis also showed that the *Msi2*^−*/*−^ mice had a significant decrease in both BV/TV and Tb.Th and also showed a significant increase in Tb.Sp but no changes in Tb.N (Fig. 3i–l). The numbers of osteoblasts per bone perimeter (N.Ob/B.Pm) and osteoblast surface per bone surface (Ob.S/BS) were decreased in the *Msi2*^−*/*−^mice compared to the WT control mice (Fig. 3m, n). Further immunofluorescence staining analysis of the distal femur of the *Msi2*^−*/*−^ mice revealed decreased expression of the osteoblast markers osteopontin (OPN) and COL1α1 in the *Msi2*^−*/*−^ mice (Fig. 3o–r).

Bone formation by osteoblasts and bone resorption by osteoclasts are essential for the maintenance of bone homeostasis. Our results showed that the osteoclast differentiation of the Msi2 knockout mice was weakened in vitro (Fig. S3A, B). Interestingly, no changes in the number of HSCs were detected in the bone marrow cells of the *Msi2*^−^^*/*−^ mice (Fig. S3C). TRAP staining for osteoclast activity showed no significant difference between the WT and *Msi2*^−*/−*^ mice in vivo (Fig. S3D, E). This finding indicates that the decrease in bone mass in the *Msi2*^−*/−*^ mice is mainly due to decreased bone formation. Taken together, the above results suggested that *Msi2* is required for proper bone formation.

### *Msi2* promotes osteoblastogenesis and inhibits BMSC adipogenesis

**Fig. 4:**
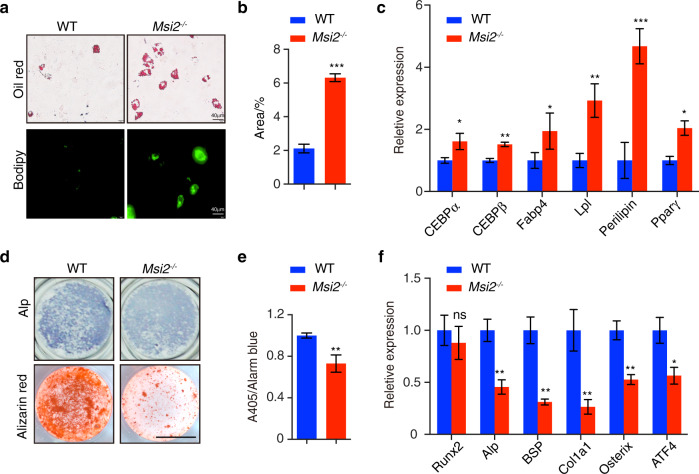
*Msi2* deficiency promotes adipogenesis and inhibits osteoblastogenesis in BMSCs. **a** Oil Red O and BODIPY staining of BMSCs cultured with adipocyte differentiation medium for 6 days. Data are representative of three independent experiments. Scale bar = 40 μm. **b** Statistical analysis of the percentage of Oil Red O-positive area via ImageJ. Data are presented as the mean ± SD, *n* = 4 in each group. Data represent the mean ± SD, ***P < 0.005, unpaired Student’s *t* test. **c** qPCR analysis of *Cebpα*, *Cebpβ*, *Fabp4*, *Lpl, perilipin* and *Pparγ* expression in BMSCs from the WT and *Msi2*^*−/−*^ mice after adipocyte differentiation for 6 days. Data represent the mean ± SD, *n* = 4. *P < 0.05, **P < 0.01, ***P < 0.005, unpaired Student’s *t* test. **d** ALP staining and Alizarin red S staining after osteoblast differentiation for 7 days (upper) and 14 days (lower). Data are representative of three independent experiments. Scale bar = 3 mm. **e** ALP activity was measured by phosphatase substrate assays. Data represent the mean ± SD, *n* = 3. **P < 0.01, unpaired Student’s *t* test. **f** qPCR analysis of *Runx2*, *Alp*, *Bsp*, *Col1α1*, *Osterix*, and *ATF4* expression after osteoblast differentiation for 7 days; BMSCs were from the WT and *Msi2*^*−/*−^ mice. Data represent the mean ± SD, *n* = 4. *P < 0.05, **P < 0.01, ns: no significance; unpaired Student’s *t* test

The accumulation of adipocytes and decreased bone formation in the bone of the *Msi2*^*−/*−^ mice prompted us to further explore how *Msi2* regulates BMSC differentiation. BMSCs were isolated from the WT or *Msi2*^*−/−*^ mice and were differentiated in adipogenic medium for 7 days. Adipogenic differentiation was enhanced in the *Msi2*^*−/*−^ BMSCs, as indicated by increased Oil Red O staining and BODIPY staining compared to that of the BMSCs from the WT mice (Fig. 4a, b). The expression levels of adipocyte markers such as *Cebpα, Cebpβ, Fabp4*, lipoprotein lipase *(Lpl), perilipin* and *Pparγ* increased in the *Msi2* knockout BMSCs upon induction to adipogenesis compared to those of the WT BMSCs (Fig. 4c).

We next examined the role of *Msi2* in the osteoblast differentiation of BMSCs. BMSCs were isolated from the WT or *Msi2*^−*/*−^ mice and were induced to differentiate in osteogenic medium for 1 week and 2 weeks. Alkaline phosphatase (ALP) activity assays and Alizarin red histochemical staining revealed reduced osteoblast differentiation in BMSCs from the *Msi2*^*−/−*^ mice (Fig. 4d, e). The expression levels of osteoblast markers, such as *Alp*, bone sialoprotein (*Bsp*), *Col1α1*, *Osterix* and *Atf4*, also decreased in the *Msi2*^*−/−*^ BMSCs (Fig. 4f).

Taken together, the above results revealed that *Msi2* regulates the balance of BMSC fate commitment by repressing adipocyte differentiation and enhancing osteoblast differentiation.

### *MSI2* inhibits PPAR signaling in BMSCs

**Fig. 5:**
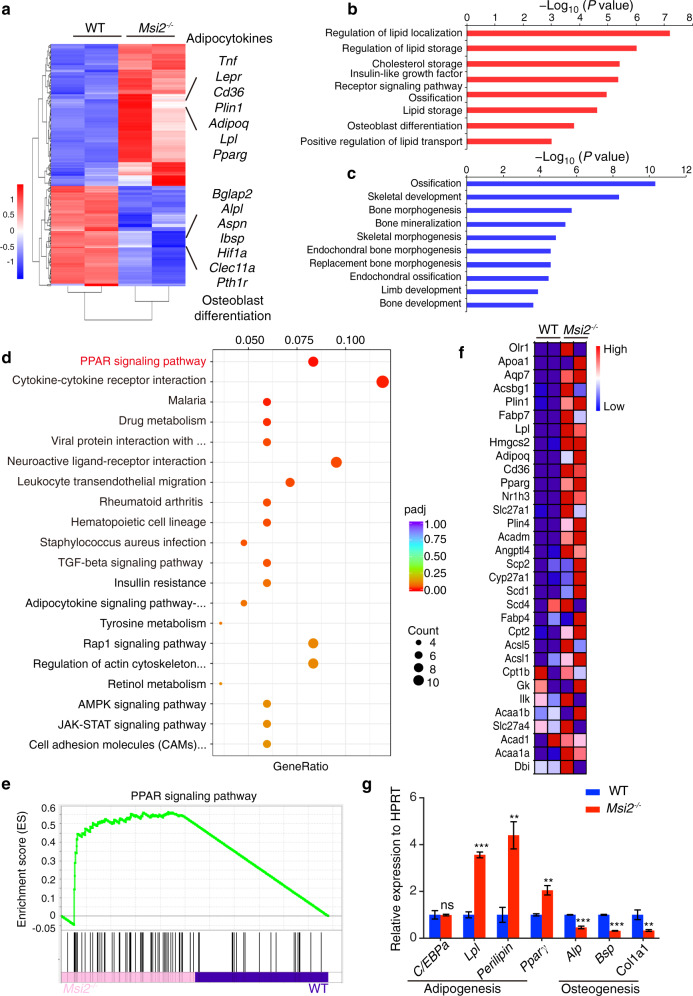
*MSI2* inhibits PPAR signaling in BMSCs. **a** Heatmap of RNA sequencing data between the WT and Msi2^−/−^ mouse BMSCs cultured in osteoblast differentiation medium for 7 days, *n* = 2 for each group. **b** Upregulated (red) GO analysis associated with significantly regulated genes (*P* < 0.05) in the Msi2 knockout versus WT control groups. **c** Downregulated (blue) GO analysis associated with significantly regulated genes (*P* < 0.05) in the Msi2 knockout versus WT control groups. **d** Upregulated (red) pathways associated with significantly regulated genes (*P* < 0.05) in the Msi2 knockout versus WT control groups. **e** GSEA of the enrichment of all genes in RNA sequencing. **f** Heatmap of genes with upregulated expression in the PPAR signaling pathway obtained by GSEA. **g** qPCR results of adipogenesis-related gene (*Cebp*α, *Lpl, Perilipin, Pparγ*) and osteogenesis-related gene (*Alp, Bsp, Col1α1*) expression in the WT and Msi2^−/−^ mouse BMSCs

To explore the molecular mechanism by which *Msi2* regulates osteoblast-adipocyte lineage commitment, we performed RNA sequencing analysis using BMSCs from the WT and *Msi2*^−*/*−^ mice (7 days after osteoblast differentiation) and compared the gene expression profiles. Genes related to adipocyte differentiation showed upregulated expression, and genes related to osteoblast differentiation showed downregulated expression (Fig. 5a). Gene set enrichment analysis (GSEA) was then performed to identify significantly enriched Gene Ontology (GO) terms. Lipid localization or storage regulators and adipocyte differentiation markers showed upregulated expression in the *Msi2*^−/−^ BMSCs (Fig. 5b). Ossification-, skeletal development- and bone development-related genes showed significantly downregulated expression (Fig. 5c). Kyoto Encyclopedia of Genes and Genomes pathway analysis indicated that the PPAR signaling pathway was significantly enhanced in the Msi2 knockout BMSCs (Fig. 5d). To further analyze the changes in the PPAR signaling pathway in the Msi2 knockout cells, we utilized GSEA to mine the RNA-seq data, and the results showed that Msi2 knockout increased the enrichment score for the PPAR signaling pathway module (Fig. 5e). Genes with upregulated expression that showed a significant difference in expression in the GSEA were visualized by a heatmap (Fig. 5f). The expression levels of the genes with upregulated and downregulated expression were further confirmed in the Msi2 knockout BMSCs by RT-PCR (Fig. 5g). As PPARγ is considered to be one of the major drivers of adipogenesis,^10,11^ these results suggested that *Msi2* may regulate BMSC commitment by inhibiting the PPARγ signaling pathway.

### *Msi2* inhibits *Cebp*α translation and PPARγ expression in BMSCs

**Fig. 6:**
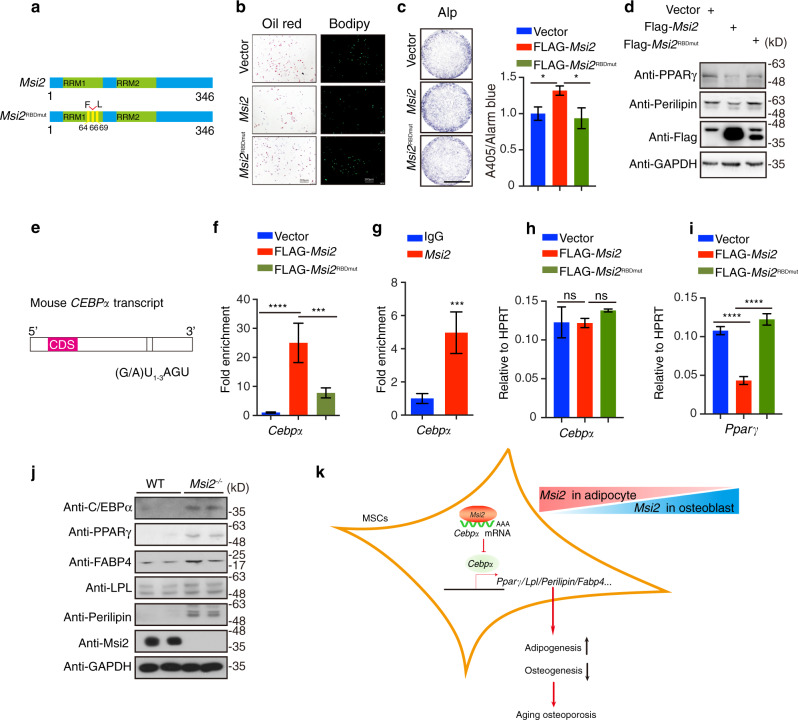
*Msi2* inhibits *Cebpα* translation and PPARγ activation in BMSCs. **a** Schematic illustration of *Msi2* and the *Msi2*^RBDmut^ mutation. **b** BMSCs isolated from 4-week-old wild-type mice and treated with *Msi2* and *Msi2*^*RBD*^ lentivirus. Cultures were stained with Oil Red O and BODIPY as shown. Scale bar = 200 μm. **c** BMSCs isolated from 4-week-old wild-type mice, and treated with *Msi2* and *Msi2*^*RBD*^ lentivirus. Cultures were stained with ALP, and ALP activity was quantified as shown. Scale bar = 3 mm. Data represent the mean ± SD, *P < 0.05, one-way ANOVA. **d** Western blot analysis of PPARγ and perilipin protein levels in the C3H10 cells overexpressing Flag-tagged *Msi2* and *Msi2*^RBDmut^ protein; GAPDH was used as a reference protein. **e** Schematic of the mouse *Cebpα* transcript. Bars, the putative MBEs (r(G/A)U_1–3_AGU). Two MBEs were identified within the 3′ UTR of *Cebpα*. CDS, coding sequence for mC/EBPα protein. **f** RIP with anti-Flag antibody from C3H10 cells expressing empty vector, Flag-tagged *Msi2* or Flag–*Msi2*^RBDmut^. Coimmunoprecipitated RNAs were analyzed for the enrichment of *Cebpα* transcripts. *n* = 3 each. Data represent the mean ± SD, ***P < 0.001, ****P < 0.000 1, one-way ANOVA. **g** RIP with anti-*Msi2* antibody or a control rabbit IgG from BMSCs. Coimmunoprecipitated RNAs were analyzed for the enrichment of *Cebpα* transcripts. *n* = 3 each. Data represent the mean ± SD, ***P < 0.001, ordinary one-way ANOVA. **h** qPCR results of *Cebpα* in the C3H10 cells overexpressing Flag-tagged *Msi2* and *Msi2*^RBDmut^ proteins. Data represent the mean ± SD, ns: no significance, one-way ANOVA. **i** qPCR results of *Pparγ* in the C3H10 cells overexpressing Flag-tagged *Msi2* and *Msi2*^RBDmut^ proteins. Data represent the mean ± SD, ****P < 0.000 1, one-way ANOVA. **j** Western blot analysis of C/EBPα, PPARγ, FABP4, LPL, perilipin and *Msi*2 protein levels in the WT and *Msi2*^−*/−*^ mouse BMSCs. GAPDH was used as a reference protein. **k** The model of *Msi2* regulating PPAR signaling

*Msi2* is an RNA-binding protein. Previous results demonstrated that three phenylalanine residues in Msi2 are essential for Msi2 RNA binding. To determine whether RNA binding is essential for the function of *Msi2*, we mutated three phenylalanine residues essential for *Msi2* RNA binding to leucine (F64/66/69 L) to generate an RNA binding-deficient mutant of *Msi2* (hereafter *Msi2*^RBDmut^) (Fig. 6a).^16,27^ We next compared the function of *Msi2* with that of *Msi2*^RBDmut^. As shown in Fig. 6b, overexpression of *Msi2* reduced the differentiation of BMSCs into adipocytes, but *Msi2*^RBDmut^ overexpression did not reduce the differentiation of BMSCs into adipocytes (Fig. 6b). Moreover, overexpression of *Msi2* enhanced the differentiation of BMSCs into osteoblasts, but *Msi2*^RBDmut^ overexpression did not (Fig. 6c). These results suggest that the mRNA binding activity of *Msi2* is required for BMSC commitment.

Transcriptional profiling analysis suggested that *Msi2* may regulate BMSC commitment by inhibiting the PPARγ signaling pathway. *Msi2* is considered to be a translational repressor by binding the 3′ UTR of the target mRNA.^13^ We next explored whether *Msi2* regulates the PPARγ signaling pathway by repressing the translation of key components of PPARγ signaling. As shown in Fig. 6d, overexpression of *Msi2* reduced the protein levels of PPARγ and perilipin when BMSCs were induced to differentiate into adipocytes (Fig. 6d). In contrast, *Msi2*^RBDmut^ overexpression abolished the inhibitory effect of *Msi2* (Fig. 6d). These results indicated that mRNA binding activity is required for *Msi2* to inhibit the PPARγ signaling pathway.

We next examined how *Msi2* relies on the mRNA binding ability to regulate PPAR signaling. The C/EBP family has been reported to be closely related to the regulation of PPAR signaling, and the mRNA level of *Cebp* factors was not changed significantly in our RNA sequencing data. We then examined the putative MBEs in the 3′ UTR of different *Cebp*s and found that only *Cebpα’s* 3′ UTR has two MBEs*;* the *Cebpβ* and *Cebpδ* 3′ UTRs did not (Fig. 6e). We then performed an RNA immunoprecipitation (RIP) assay using C3H10 cells transfected with plasmids expressing Flag-tagged *Msi2* or Flag-tagged *Msi2*^*RBDmut*^. Interestingly, *Cebpα* transcripts were significantly enriched by Flag immunoprecipitation when Flag-*Msi2* was expressed. In contrast, *Cebpα* transcripts were not enriched when Flag-tagged *Msi2*^*RBDmut*^ was expressed (Fig. 6f). These results suggested that *Msi2* binds to the mRNA of *Cebpα*. Consistently, RIP with an anti-*Msi2* antibody also specifically enriched *Cebpα* transcripts relative to that of an immunoglobulin-G (IgG) control (Fig. 6g), further confirming the interaction between *Msi2* and *Cebpα* mRNA. *Msi2* overexpression in C3H10 cells did not change the RNA level of *Cebpα* (Fig. 6h), However, the RNA level of *Pparγ*, which is regulated by *Cebpα*, was significantly downregulated when *Msi2* was overexpressed in C3H10 cells (Fig. 6i). The protein level of *Cebp*α was increased significantly in the MSI2 knockout BMSCs, and PPARγ signaling markers were also significantly increased in the MSI2 knockout BMSCs (Fig. 6j). These data indicated that binding of *Msi2* to *Cebpα* transcripts negatively regulates the translation of *Cebpα*. Regulation of PPARγ signaling by *Msi2* is essential for the dynamic balance of the commitment between osteoblasts and adipocytes (Fig. 6k).

### *Msi2* expression is downregulated during aging

**Fig. 7:**
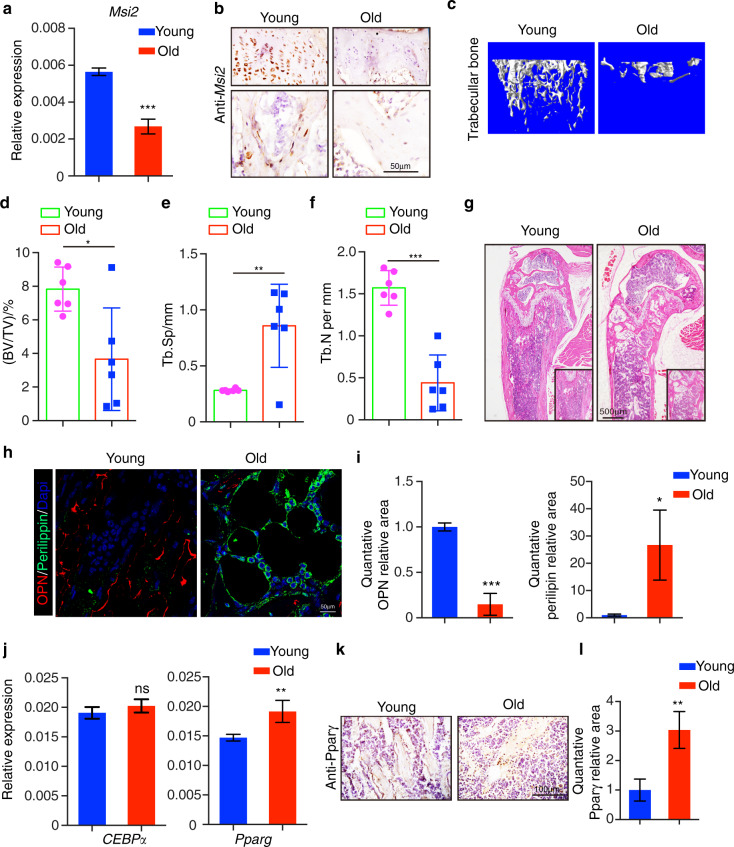
*Msi2* expression is downregulated during aging. **a** qPCR results of *Msi2* expression in BMSCs from 8-week-old and 24-month-old mice (*n* = 4). Data represent the mean ± SD, ***P < 0.001, unpaired Student’s *t* test. **b** Immunohistochemistry staining of *Msi2* from 8-week-old and 24-month-old mice. Scale bar = 50 μm. **c** Three-dimensional μ-CT images of trabecular bone of distal femurs isolated from 8-week-old female and 24-month-old female mice (*n* = 6). **d** μ-CT analysis of trabecular bone volume per tissue volume (BV/TV) in the distal femur of 8-week-old female and 24-month-old female mice. **e** μ-CT analysis of the distal femur of 8-week-old female and 24-month-old female mice for trabecular separation (Tb.Sp). **f** μ-CT analysis of the trabecular number (Tb.N) of the distal femur of 8-week-old female and 24-month-old female mice. **g** Hematoxylin-eosin staining of femurs from wild-type mice at 8 weeks and 24 months. Scale bar = 500 μm. **h** Immunostaining of perilipin A/B (green) and OPN (red) of femurs from 8-week-old and 24-month-old mice. Scale bar = 50 μm. **i** Quantification of the relative areas of OPN and Perilipin in (**h**). **j** qPCR results of *Cebpα* and *Pparγ* expression in BMSCs from 8-week-old and 24-month-old mice (*n* = 4). Data represent the mean ± SD, ***P < 0.01, ns: no significance, unpaired Student *t* test. **k** Immunohistochemistry staining of PPARγ from 8-week-old and 24-month-old mice. Scale bar = 100 μm. **l** Quantification of the relative area of PPARγ in (**k**)

The depletion of *Msi2* in mice led to decreased bone mass with increased marrow adipocytes, resembling aging-induced osteoporosis. We next examined whether *Msi2* expression changed during aging. We isolated BMSCs from 2-month-old (young) or 24-month-old (old) mice and found that the *Msi2* expression level was decreased in old BMSCs, as indicated by RT-qPCR assays (Fig. 7a). Immunohistochemical staining also showed that *Msi2* protein expression levels were downregulated in the aged mice (Fig. 7b). μ-CT analysis confirmed that the bone mass of the old mice was significantly reduced (Fig. 7c, d), accompanied by increased Tb.Sp (Fig. 7e) and decreased Tb.N (Fig. 7f). Interestingly, compared with that in the young mice, cortical bone in the aging mice was thicker (Fig. S4A, B). Similar to the phenotype of the *Msi2* knockout mice, abnormal accumulation of adipocytes in the bone marrow cavity of the aged mice was observed (Fig. 7g), suggesting the occurrence of aging-related osteoporosis. Immunofluorescence staining revealed decreased expression levels of the osteoblast marker OPN and increased expression levels of the adipocyte marker perilipin in the bone marrow cavity of the old mice (Fig. 7h, i). Similar to the scenario in *Msi2*^−/−^ BMSCs, the RNA level of *Cebpα* remained unchanged in BMSCs isolated from the aged mice, and the RNA level of *Pparγ* increased in BMSCs isolated from the aged mice (Fig. 7j). Immunohistochemical staining also showed that PPARγ protein expression levels were upregulated in the aged mice (Fig. 7k, l). Consistent with previous reports,^28^ the mRNA level of the senescence marker *p16* increased in the old BMSCs. In addition, the target genes of PPARγ increased significantly (Fig. S4C). The old BMSCs had a phenotype similar to that of the *Msi2*^−*/−*^ BMSCs, which is consistent with the decreased expression level of *Msi2* in the aged BMSCs.

These results suggest that *Msi2* could be one of the contributors to aging-induced osteoporosis. In old BMSCs, the reduction in the *Msi2* expression level leads to a shift in the differentiation balance of BMSCs. Adipogenesis is enhanced, and osteoblastogenesis declines, which results in aging-induced osteoporosis.

## Discussion

RNA-binding proteins play an important role in cell fate determination through posttranscriptional regulation. Here, we found that the RNA-binding protein *Msi2* controls the fate of BMSCs. By binding to the 3′ UTR of the mRNA of the key adipogenesis-related factor, *Msi2* inhibits the adipogenic potential of BMSCs. In aged BMSCs, the *Msi2* expression level decreased, and the balance of BMSC differentiation shifted toward adipogenesis, which led to osteoporosis indicated by abnormal accumulation of adipocytes in the bone marrow cavity and decreased bone mass. Our results revealed that *Msi2* is an important contributor to osteoporosis by modulating protein translation.

MSI is an evolutionarily conserved family of RNA-binding proteins that play key roles in the maintenance of self-renewal of stem cells and HSC fate.^15,29^ Previous studies on *Msi2* have mainly focused on its function in tumors.^30–32^ In this study, we reported for the first time that *Msi2* regulates BMSC commitment. *Msi2* plays a key role in maintaining the balance between osteoblastogenesis and adipogenesis. Although several previous studies have shown that there is a negative correlation between MSC osteogenesis and adipogenesis,^3,4,33,34^ the RNA-binding protein involved is the first to be discovered. Among the limited number of genes identified downstream of MSI2,^13,19,27^ our study found that MSI2 specifically binds to the 3′ UTR of *Cebpα* to regulate PPARγ signaling and control the differentiation of BMSCs. These results suggest that RNA-binding proteins such as *Msi2* can be considered upstream of the PPARγ signaling pathway for drug targeting research.

Aging-related osteoporosis causes progressive fat accumulation and trabecular bone loss.^35^ Existing studies on osteoporosis have mainly focused on transcription factors, epigenetics and hormone metabolism.^4,36,37^ However, the mechanism of RNA-binding proteins involved in regulating osteoporosis remains to be discovered. In our study, it was confirmed that *Msi2* greatly reduced both RNA and protein levels in aging mouse bone samples, and the*Msi2*-deficient mice showed an age-dependent osteoporosis-like phenotype. Our research established a link between osteoporosis and RNA-binding proteins, and we discovered a regulatory relationship between MSI2 and PPARγ signaling. This result may provide new ideas for future research on targeted therapy for osteoporosis.

There are several limitations in our study. The animal model used in this study is *Msi2*^*−/*−^ mice, and the influence of other organs on bone cannot be ruled out. Although the expression level of *Msi2* in the BMSCs was not the highest, we found that the expression level of *Msi2* in the spleen was lower than that in the BMSCs, and *Msi2* has been shown to play an important role in the spleen. Our knockout mice also have a similar phenotype of reduced spleen.^19^ We observed a certain difference between the detection of RNA levels and the detection of protein levels, which may be caused by slightly different posttranscriptional translation of proteins in different tissue environments. However, in vivo and in vitro experiments showed that *Msi2* was knocked out in BMSCs, which resulted in an osteoporotic phenotype. Although we have established a connection between *Msi2* and PPARγ signaling, the direct genetic evidence remains to be further examined.

Our work reveals the function of *Msi2* in regulating the commitment of MSCs, thereby regulating bone homeostasis. We further tested the proportion of HSCs in the *Msi2*^−*/*−^ mice and the control mice, and the results showed that the absence of *Msi2* did not affect the proportion of HSCs in bone marrow cells (Fig. S3C). Although we also observed that Msi2 deletion inhibited osteoclast differentiation in vitro,^25^ there was no significant difference in TRAP staining in vivo. The effect of *Msi2* on bone in HSCs through other methods of compensation cannot be ruled out. This issue also needs to be revealed in future research.

Because RNA sequencing showed the tight integration of the *Msi2* and PPAR signaling pathways, *Msi2* plays a key role in the regulation of the PPAR signaling pathway in mesenchymal stem cells. We focused on genes such as *Cebpα, Cebpβ, Pparg, Fabp4, and Lpl*. We excluded some genes by analyzing whether there are Msi2 binding sites on the 3′ UTR and then conducted RIP verification, but unfortunately, the results were not verified by the RIP experiment. In addition, we focused on *Runx2*, which is the core transcription factor for bone formation. However, we did not find a mouse skull closure disorder, and the results were not verified in the RIP experiment. We do not know whether Msi2 will bind to the 3′ UTR of other molecules and regulate protein expression, nor can we eliminate other ways of *Msi2* molecular regulation. Future work should investigate these issues.

RNA-binding proteins are closely related to the occurrence and development of cancer. As a potential target for cancer treatment, small molecules have been developed to act as inhibitors of *Msi2*. We need to consider the effect of this medication on patients with osteoporosis and other skeletal degenerative diseases.^38,39^ Realizing tissue-specific and spatiotemporal specificity to restore the normal expression of *Msi2* will be crucial for the occurrence and development of the disease. Exploring compounds and small molecules that regulate *Msi2* will promote the treatment of cancer and osteoporosis.

Overall, our work demonstrated that *Msi2* functions as a repressor of *Cebpα* to inhibit the activation of PPARγ signaling. This work defined the role of *Msi2* in regulating MSC commitment and identified a new target for aging-induced osteoporosis treatment. It is not clear whether *Msi2* is also involved in regulating the translation of other targets in the process of aging. If so, how *Msi2* cooperates among different molecules will be another interesting question worthy of further study.

## Materials and methods

Msi2 mice were constructed using the Crispr-Cas9 strategy. *Msi2*^*−/−*^ mice were constructed using the Crispr-Cas9 strategy. At the end of the first exon of *Msi2*, 10 bases were deleted using Crispr-Cas9 technology, resulting in a gene frameshift. The deleted base sequence is AGCACGACCC. All mice analyzed had a C57BL/6 background. Animals were maintained under specific pathogen-free conditions in the institutional animal facility of the Shanghai Institute of Biochemistry and Cell Biology, Chinese Academy of Sciences. All animal experiments were performed with a protocol approved by the Animal Care and Use Committee of Shanghai Institute of Biochemistry and Cell Biology, Chinese Academy of Sciences.

### Antibodies

Anti-Flag antibody (F-3165, 1:5 000, Sigma), rabbit IgG (SC-2027, Santa Cruz Biotechnology), anti-perilipin A/B (Sigma, P1873), and anti-OPN (R&D, AF808) were used. Anti-Col1a1 (Rockland, 600-400-103), anti-PPARγ (Santa Cruz, sc-7273), anti-LPL (R&D, AF7197) and anti-*Msi2* (Abcam, ab76148) were obtained.

### Cell culture

Cells were cultured at 37 °C in humidified incubators containing an atmosphere of 5% CO_2_. HEK-293T cells were maintained in DMEM (Corning, Corning, NY) supplemented with 10% fetal bovine serum (FBS) and 1% penicillin/streptomycin (Gibco) solution. C3H10T1/2 cells were maintained in α-MEM (Corning, Corning, NY) supplemented with 10% FBS and 1% penicillin/streptomycin (Gibco) solution.

### Osteoblast differentiation and adipocyte differentiation

We collected femurs from the WT and *Msi2*^*−/*−^ mice and flushed out the bone marrow cells with phosphate-buffered saline (PBS). All nuclear cells were seeded (2 × 10^6^ cells per dish) in 100 mm culture dishes (Corning) and incubated at 37 °C under 5% CO_2_ conditions. After 24 h, the cells were supplemented with fresh medium. After 48 h, nonadherent cells were washed with PBS, and adherent cells were cultured in alpha minimum essential medium (Corning, Corning, NY) supplemented with 10% FBS and 1% penicillin/streptomycin (Gibco) solution for an additional 5 days.

For induction of the differentiation of BMSCs into adipocytes, cells were first cultured in adipogenic induction medium (α-MEM/10% FBS containing 1 μmol·L^−1^ dexamethasone, 0.1 mmol^.^L^−1^ rosiglitazone, 0.5 mmol·L^−1^ IBMX, 10 µg^.^mL^−1^ insulin) for 1 day, and then, adipogenic maintenance medium (α-MEM/10% FBS containing 10 µg^.^mL^−1^ insulin) was added for 2 days. After mature adipocyte formation, cells were stained with 2 mg^.^mL^−1^ Oil Red O solution or BODIPY 493/503.

For induction of the differentiation of BMSCs into osteoblasts, cells were cultured in α-MEM containing 10% FBS, 50 μg^.^mL^−1^ L-ascorbic acid, and 1080 mg^.^mL^−1^ β-glycerophosphate. The osteoblast differentiation assay was performed following a previously published method. For quantitative analysis of ALP activity, cells were incubated with Alamar Blue to calculate cell numbers and then incubated with phosphatase substrate (Sigma-Aldrich, St. Louis, MO) dissolved in 6.5 mmol^.^L^−1^ Na_2_CO_3_, 18.5 mmol^.^L^−1^ NaHCO_3_, and 2 mmol^.^L^−1^ MgCl_2_ after washing with PBS. ALP activity was then read with a luminometer (Envision). Bone nodule formation was stained with 1 mg^.^mL^−1^ Alizarin red S solution (pH 5.5) after 14 days of induction.

### RIP assay

BMSCs isolated from the WT and *Msi2*^*−/*−^ mice or C3H10 cells infected with Flag-tagged *Msi2* or Flag-tagged *Msi2*^RBDmut^ lentivirus were lysed in 50 mmol^.^L^−1^ Tris/HCl (pH 7.4) containing 100 mmol^.^L^−1^ NaCl, 5 mmol^.^L^−1^ EDTA, 1% NP-40, Protease Inhibitor Cocktail (HY-K0010, 1:100, MedChem Express), and RNase inhibitor (Thermo Scientific). We performed immunoprecipitations with anti-FLAG, anti-*Msi2* or rabbit normal IgG and protein G magnetic beads for 2 h at 4 °C. The immunoprecipitated protein-RNA complexes were washed five times with wash buffers (25 mmol^.^L^−1^ Tris/HCl (pH 7.4), 20 mmol^.^L^−1^ MgCl_2_, 100 mmol^.^L^−1^ NaCl, 0.2% Tween-20, and 0.05% NP40). Total RNA was purified from the washed beads using TRIzol (T9424, Sigma) and subjected to RT-qPCR analysis for quantification. For each sample, we calculated the enrichment multiple of the transcript content in the RIP score relative to the amount (RIP/input) that was present before the RIP in the input sample.

### RNA-seq

Isolation of total RNA with TRIzol comes from samples of osteoblastic differentiation induced by BMSCs from the WT and *Msi2*-deficient mice for 7 days. RNA library construction, sequencing and analysis are provided by Novogene. The top GO categories were selected according to the *P* values.

### Real-time RT-PCR analysis

Total RNA was isolated from cells with TRIzol reagent (T9424, Sigma), and first-strand cDNA was synthesized from 500 ng of total RNA using the PrimeScript^™^ RT Reagent Kit (PR037A, TaKaRa). Real-time reverse transcriptase RT-PCR was performed with the Bio-Rad CFX96 system. Gene expression from RT-PCR was quantified relative to that of Hprt or Gapdh.

### IP and immunoblotting

First, 293 T cells were seeded at 1 – 2 × 10^7^ cells per 10 cm dish and cultured overnight. After transfection with PEI for 48 h, the cells were harvested and washed with cold PBS following experimental treatments. Then, the cells were lysed with EBC buffer (50 mmol^.^L^−1^ Tris, pH 7.5, 120 mmol^.^L^−1^ NaCl and 0.5% NP-40) containing protease inhibitor cocktail (HY-K0010, 1:100, MedChem Express). After ultrasonication (power: 25%, sonicate 5 s, stop 5 s, five times), lysates were subjected to IP with anti-Flag beads (M2, Sigma) at 4 °C for 4–6 h or overnight, followed by washing in lysis buffer, SDS–PAGE electrophoresis and immunoblotting with the indicated antibody.

### μ-QCT analysis

Preparation of skeletal tissue and μ-QCT analysis were performed as previously described.^3^ The mouse femurs isolated from age- and sex-matched mice were skinned and fixed in 70% ethanol. Scanning was performed with the instrument μ-QCT system SkyScan1176 (Bruker Biospin). The mouse femurs were scanned at a 9 μm resolution for quantitative analysis. Three-dimensional images were reconstructed using a fixed threshold.

### Histology and immunofluorescence

Tissues were fixed in 4% paraformaldehyde for 48 h, incubated in 15% DEPC-EDTA (pH 7.8) and ultrasonically decalcified. The specimens were embedded in paraffin or OCT and cut into 7 μm sections.

Immunofluorescence assay: Sections were blocked in PBS with 10% horse serum and 0.1% Triton for 1 h at room temperature. Then, the cells were stained overnight with rabbit anti-perilipin A/B (Sigma, P1873, 1:1 000, USA) and OPN (1:1 000; R&D, AF808). Donkey-anti-rabbit Alexa Fluor 488 (1:1 000; Molecular Probes, A21206) and donkey-anti-goat Cy3 (1:1 000; Jackson ImmunoResearch, 705–165–147) were used as secondary antibodies. DAPI (Sigma, D8417) was used for counterstaining. Slides were mounted with anti-fluorescence mounting medium (Dako, S3023), and images were acquired with an Olympus FV3000 and SP8 confocal microscope.

Immunohistochemical staining and Col1a1 (1:100; Rockland, 600–400–103) staining were performed as described by Dako.

Tissue sections were used for TRAP, BODIPY, and Oil Red O staining according to the standard protocol.

### Statistics

Statistical analysis was performed by unpaired, two-tailed Student’s *t* test for comparison between two groups using GraphPad Prism Software. Through Prism software, one-way ANOVA was used to compare and analyze the three groups of data. A *P* value of <0.05 was considered statistically significant.

## Supplementary information

supplementary Figure1

supplementary Figure2

supplementary Figure3

supplementary Figure4
