# Supplementary figures and images for: The RNA-binding protein Musashi2 governs osteoblast-adipocyte lineage commitment by suppressing PPARγ signaling
Source: Bone Res. 2022 Mar 17;10:31. doi: 10.1038/s41413-022-00202-3 (PMC8930990; doi:10.1038/s41413-022-00202-3)

# Supplymentary Figure 1

A

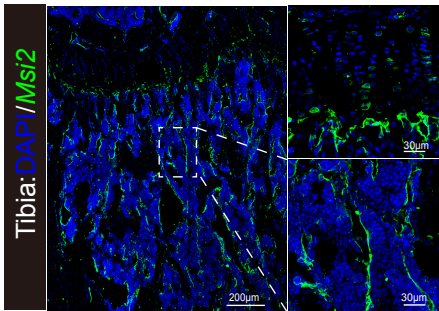

B

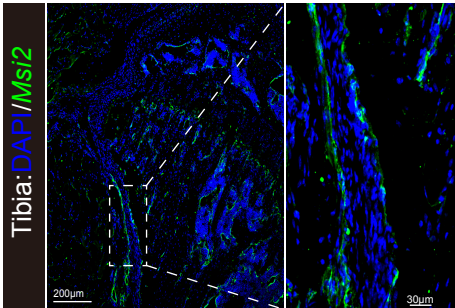

Supplement: Supplementary file 1 — supplementary Figure1 [file 41413_2022_202_MOESM1_ESM.pdf]

# Supplementary Figure 2

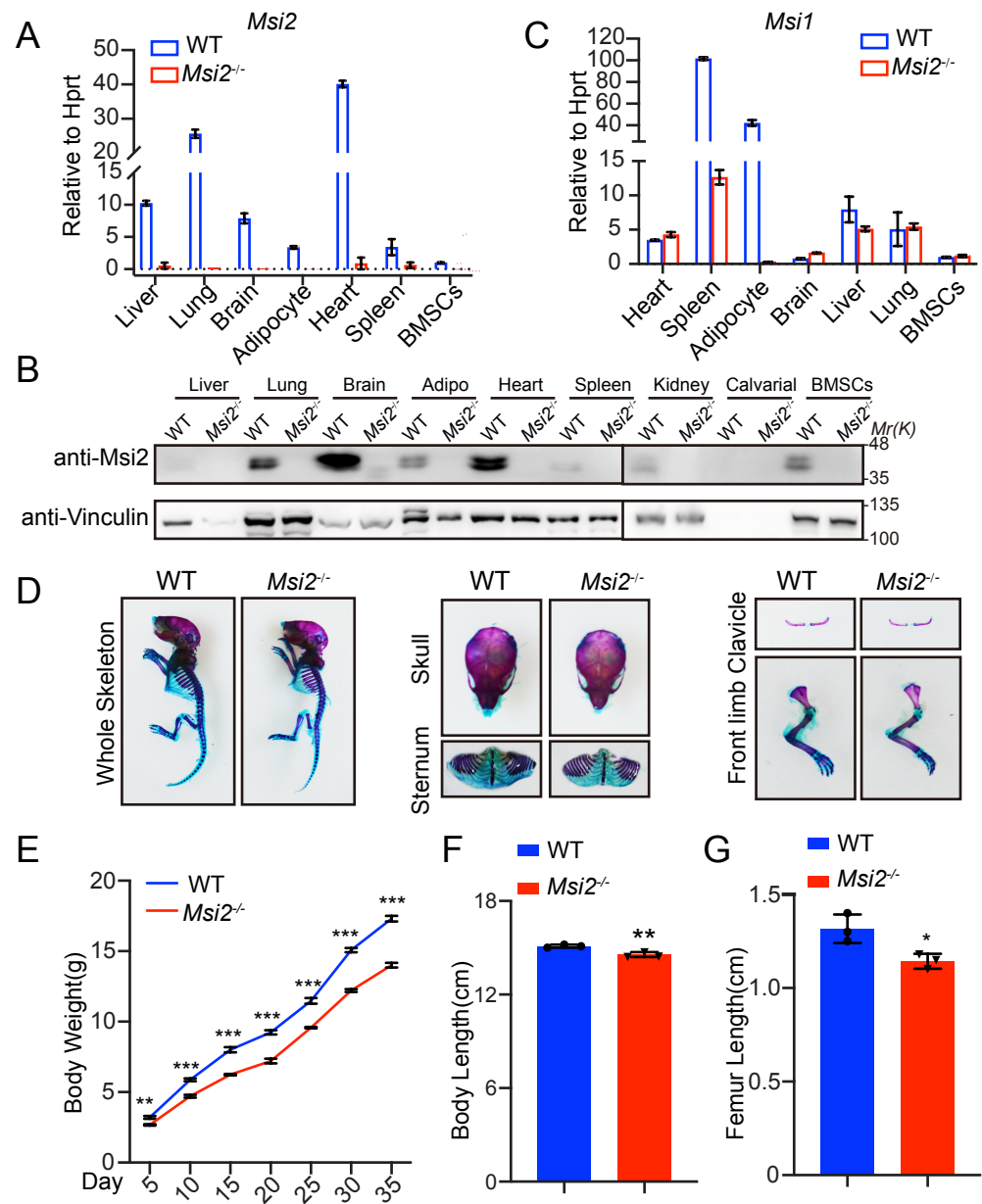

Supplement: Supplementary file 2 — supplementary Figure2 [file 41413_2022_202_MOESM2_ESM.pdf]

# Supplementary Figure 3

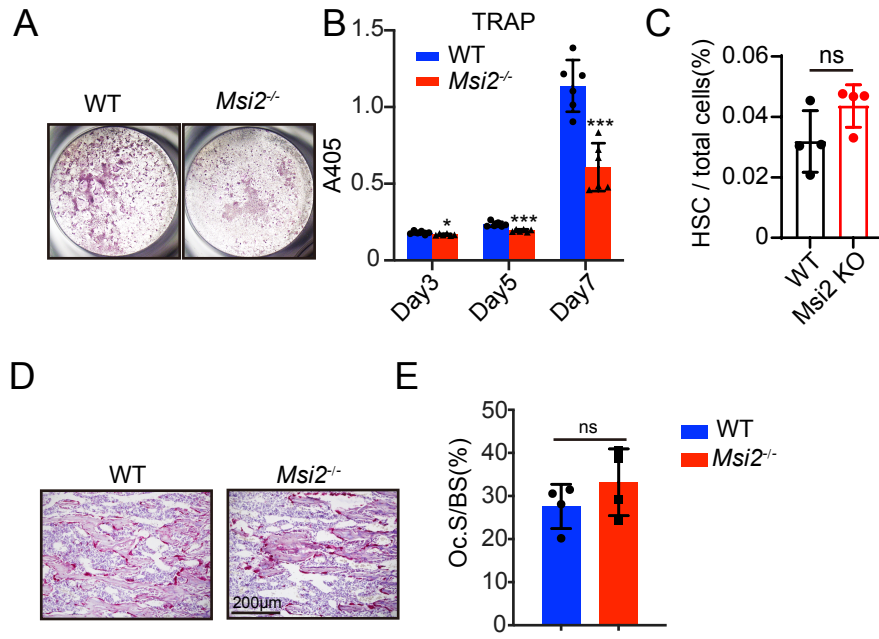

Supplement: Supplementary file 3 — supplementary Figure3 [file 41413_2022_202_MOESM3_ESM.pdf]

## Supplymentary Figure 4

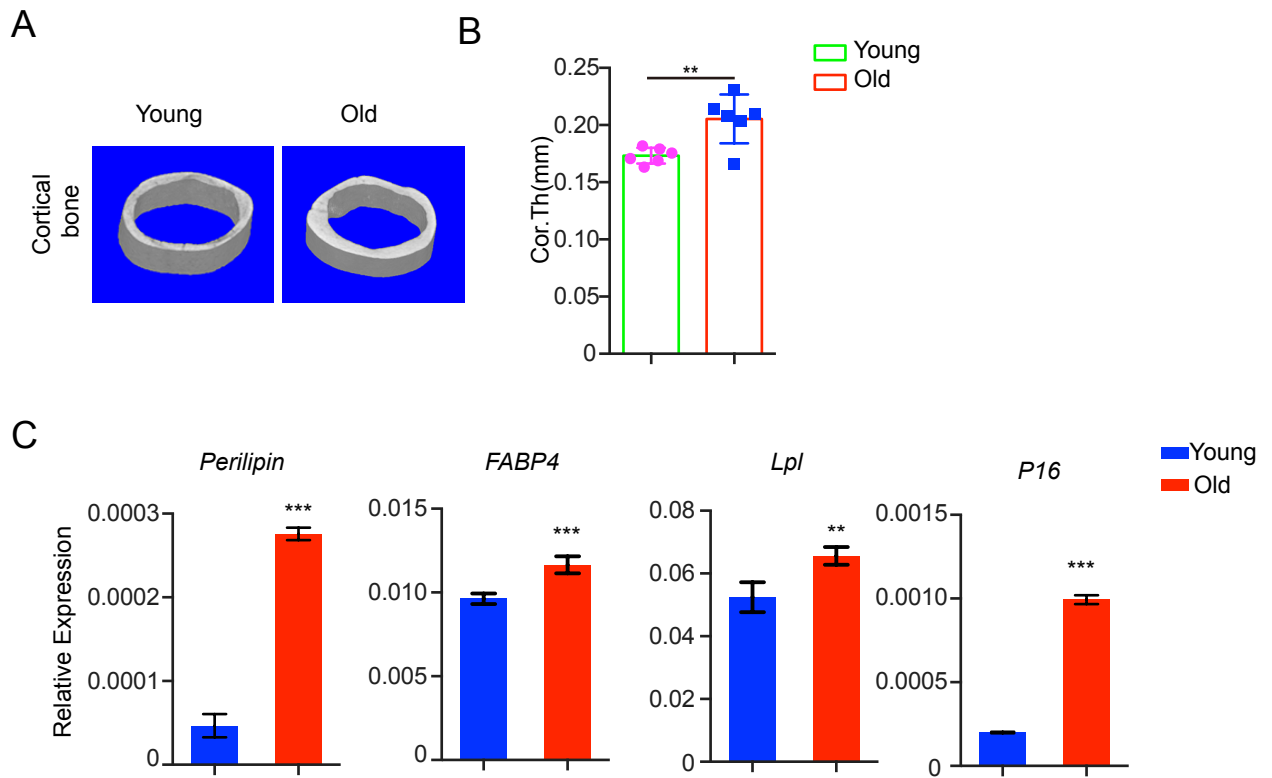

Supplement: Supplementary file 4 — supplementary Figure4 [file 41413_2022_202_MOESM4_ESM.pdf]
